# Aberrant Plasma Cell Contamination of Peripheral Blood Stem Cell Autografts, Assessed by Next-Generation Flow Cytometry, Is a Negative Predictor for Deep Response Post Autologous Transplantation in Multiple Myeloma; A Prospective Study in 199 Patients

**DOI:** 10.3390/cancers13164047

**Published:** 2021-08-11

**Authors:** Ioannis V. Kostopoulos, Evangelos Eleutherakis-Papaiakovou, Pantelis Rousakis, Ioannis Ntanasis-Stathopoulos, Chrysanthi Panteli, Nikolaos Orologas-Stavrou, Nikolaos Kanellias, Panagiotis Malandrakis, Christine-Ivy Liacos, Nikos E. Papaioannou, Aristea-Maria Papanota, Magdalini Migkou, Despina Fotiou, Maria Gavriatopoulou, Nikolaos V. Angelis, Efstathios Kastritis, Meletios-Athanasios Dimopoulos, Ourania E. Tsitsilonis, Evangelos Terpos

**Affiliations:** 1 Department of Biology, School of Science, National and Kapodistrian University of Athens, 15784 Athens, Greece; ivkostop@biol.uoa.gr (I.V.K.); prousakis@biol.uoa.gr (P.R.); cpanteli@biol.uoa.gr (C.P.); norologas@med.uoa.gr (N.O.-S.); nikppj@biol.uoa.gr (N.E.P.); nangelis@biol.uoa.gr (N.V.A.); 2 Department of Clinical Therapeutics, School of Medicine, National and Kapodistrian University of Athens, 11528 Athens, Greece; evelepapa@med.uoa.gr (E.E.-P.); johnntanasis@med.uoa.gr (I.N.-S.); nkanellias@med.uoa.gr (N.K.); panosmalan@med.uoa.gr (P.M.); liakou@med.uoa.gr (C.-I.L.); ampapanota@yahoo.gr (A.-M.P.); mmigkou@med.uoa.gr (M.M.); desfotiou@med.uoa.gr (D.F.); mgavria@med.uoa.gr (M.G.); ekastritis@med.uoa.gr (E.K.); mdimop@med.uoa.gr (M.-A.D.)

**Keywords:** Multiple Myeloma, autologous stem cell transplantation, stem cell grafts, minimal residual disease, next-generation flow cytometry, tumor microenvironment

## Abstract

**Simple Summary:**

Autologous stem cell transplantation (ASCT) remains the standard of care for transplant-eligible newly diagnosed Multiple Myeloma (NDMM) patients. However, despite its overall benefit, patients undergo various clinical outcomes post ASCT. Therefore, the identification of biomarkers that could explain, at least partially, this heterogeneity is of utmost clinical significance. The aim of this study was to assess clonal plasma cell contamination of the stem cell grafts of NDMM and evaluate its potency as a predictive/prognostic marker. Our results showed that worse responses to induction therapy correlate with higher levels of graft contamination. Importantly, our data revealed the significantly higher risk of delaying or not achieving complete remission and minimal residual disease (MRD)-negative responses among patients with graft contamination. Graft contamination is emerging as a promising predictive biomarker of clinical relevance that could be used to stratify patients post ASCT.

**Abstract:**

High-dose chemotherapy with autologous stem cell support (ASCT) is the standard of care for eligible newly diagnosed Multiple Myeloma (MM) patients. Stem cell graft contamination by aberrant plasma cells (APCs) has been considered a possible predictive marker of subsequent clinical outcome, but the limited reports to date present unclear conclusions. We prospectively estimated the frequency of graft contamination using highly sensitive next-generation flow cytometry and evaluated its clinical impact in 199 myeloma patients who underwent an ASCT. Contamination (con+) was detected in 79/199 patients at a median level 2 × 10^−5^. Its presence and levels were correlated with response to induction treatment, with 94%, 71% and 43% achieving CR, VGPR and PR, respectively. Importantly, con+ grafts conferred 2-fold and 2.8-fold higher patient-risk of not achieving or delaying reaching CR (4 vs. 11 months) and MRD negativity (5 vs. 18 months) post ASCT, respectively. Our data also provide evidence of a potentially skewed bone marrow (BM) reconstitution due to unpurged grafts, since con+ derived BM had significantly higher prevalence of memory B cells. These data, together with the absence of significant associations with baseline clinical features, highlight graft contamination as a potential biomarker with independent prognostic value for deeper responses, including MRD negativity. Longer follow-up will reveal if this corresponds to PFS or OS advantage.

## 1. Introduction

Multiple Myeloma (MM) is a relatively common plasma cell neoplasm that accounts for approximately 10% of all hematological malignancies [1,2]. Despite therapeutic advances and the emergence of novel agents with clear survival benefit, MM remains an incurable disease. The standard of care for eligible young and fit elderly newly diagnosed MM patients is currently a bortezomib-based induction treatment (usually bortezomib, lenalidomide, dexamethasone (VRD) or daratumumab, bortezomib, thalidomide, dexamethasone (Dara-VTD)) to decrease tumor burden, followed by a single or tandem high-dose melphalan (HDM) with autologous stem cell transplantation (ASCT) and lenalidomide maintenance [3]. The incorporation of novel agents as part of pre-transplant, post-transplant consolidation and maintenance regimens has substantially increased the long-term survival rates for MM patients undergoing ASCT [4,5,6,7]. However, the duration of relapse-free periods varies among patients, thus highlighting the need for clinically relevant biomarkers with a strong predictive value, especially in MM patients with high-risk features [8,9].

Autologous grafts may contain variable numbers of aberrant plasma cells (APCs) as a result of an incomplete eradication from pre-transplant therapy, and this contamination has been implied as a possible cause for early relapse after ASCT [10,11,12]. However, extensive studies have not been performed to address this point and limited available data have led to contradicting results, possibly due to variations in the sensitivity of applied methods to detect APCs in autologous apheresis products.

The presence of myeloma cells in autologous grafts may also have an impact in the reconstitution of the post-transplant bone marrow (BM) niche. Currently, there is sufficient evidence of a constant and dynamic interplay between myeloma cells and their microenvironmental components, which include mechanisms of myeloma cell survival and proliferation, drug resistance, immune escape but also anti-myeloma reactive responses [13,14]. The balance between these interactions may be crucial for maintaining myeloma cells at a minimum and controllable level, while minute disruptions of this crosstalk may lead to a progressive state [15,16].

In this context, we prospectively analyzed a large series of ASCT-eligible MM patients, focusing on the presence of myeloma contamination in autologous grafts and its impact on the subsequent clinical outcome. Our data reveal significant correlations between stem cell graft contamination and the depth of response achieved post ASCT and provide some preliminary evidence that the presence of even small numbers of residual APCs in the graft may lead to changes in BM reconstitution.

## 2. Materials and Methods

### 2.1. Patients Enrolment

The study included the prospective analysis of all eligible MM patients who were diagnosed, treated and received an ASCT at the Department of Clinical Therapeutics of the National and Kapodistrian University of Athens in Alexandra General Hospital, between April 2016 and March 2021. All patients underwent high-dose melphalan (HDM)/ASCT post induction treatment and were evaluated for the presence of APCs in their autologous stem cell apheresis collections.

All study procedures were carried out in accordance with the declaration of Helsinki (18th World Medical Association Assembly), its subsequent amendments, the Greek regulations and guidelines, as well as the Good Clinical Practice Guidelines (GCP) as defined by the International Conference of Harmonization. The study was approved by the local ethic committee of Alexandra General Hospital. All patients were informed of the purposes of the study, prior to sampling, and signed the respective informed consent.

### 2.2. ASCT Protocol

All patients who received HDM with ASCT, after achieving at least SD post upfront induction regimen, participated in this study. Patients received chemo-mobilization with cyclophosphamide and granulocyte colony-stimulating factor (G-CSF). All patients underwent peripheral blood stem cell collection with the Spectra Optia apheresis system. The minimum CD34+ stem cell dose considered sufficient for successful engraftment was 3 × 10^6^ CD34+ cells/kg. The optimal apheresis target dose was 5 × 10^6^ CD34+/kg. In case of suboptimal stem cell mobilization and collection, plerixafor was used to overcome poor stem cell mobilization.

All patients received high-dose therapy with HDM (200 mg/m^2^ or 140 mg/m^2^ for patients with impaired renal function) as conditioning regimen. Stem cell re-infusion was performed at least 24 h after the last day of collection. Patients were hospitalized during the transplantation procedure until hematopoietic recovery. During hospitalization, patients were supported with blood or platelet (PLT) transfusions, as necessary. In case of development of neutropenic fever, an empirical antibiotic regimen was administered and later modified, individually, according to microbial culture results and sensitivity data. All patients received post transplantation maintenance therapy.

### 2.3. Evaluation of Stem Cell Graft Contamination with NGF

The presence of APCs in stem cell grafts was examined with next-generation flow cytometry (NGF) following the EuroFlow guidelines for the detection of minimal residual disease (MRD) in MM. The apheresis products obtained were processed with the bulk lysis protocol and acquired cells were stained with the two established 8-color NGF panels, both containing the combinations CD19-PECy7, CD27-BV510, CD38-FITC, CD45-PerCPCy5.5, CD56-PE and CD138-BV421, with CD117-APC and CD81-APCH7 included in the surface tube and cytoplasmic kappa-APC and lambda-APCH7 in the intracellular-stained tube. Ten million cells were stained per tube and a minimum of five million events were recorded for further analysis. Acquisition of the samples was performed on a 3-laser BD FACSCantoII (BD Bioscience, San Jose, CA, USA) with a forward scatter (FSC) threshold set on 10,000. The reproducibility of the cytometric assessment was achieved following the EuroFlow standard operating procedures (SOP) for instrument set-up. The optimal PMT voltages were set with Rainbow beads (Spherotech Inc, Lake Forest, IL, USA) and daily performance was monitored with both CS&T (BD, San Jose, CA, USA) and Rainbow beads.

The analysis of recorded samples was performed using the Infinicyt^®^ software (Cytognos S.L., Salamanka, Spain). A sample was considered contaminated when a minimum number of 20 cells with the same aberrant phenotypic characteristics and the same light chain restriction could be identified. Principal component analysis of an unsupervised automatic population separator (APS) diagram that included the 8 surface markers was used for the calculation of the relative significance of each marker for the discrimination of APCs from the total nucleated compartment.

### 2.4. Analysis of the BM Niche Reconstitution

The BM niche profiling was examined for each patient who achieved CR on day 100 post ASCT using the NGF panels. A total of 17 BM subsets were characterized for each patient, which, beyond plasma cells, included B cells and their relative compartments (naïve, memory and B cell precursors), T cells and their CD27+ compartment, NK/NKT cells and their CD27+ compartment, erythroblasts, erythroid and myeloid progenitors, neutrophils, basophils, monocytes/tumor associated-macrophages, eosinophils and mast cells. The phenotypic discrimination of these subsets was described in detail elsewhere [17]. Analysis was conducted manually by two independent experts with minor variations in the relative subset distributions obtained. The optimal protein expression cut-off points used during the phenotypic discrimination of each subset were selected on the basis of internal positive and negative expression of each marker in the various other BM subsets with an established expression pattern.

### 2.5. Statistical Analysis

The statistical analyses were performed with SPSS V25.0 (IBM, Armonk, NY, USA) and a *p* value < 0.05 was considered significant for all associations. Fisher’s exact test was used to test for non-random correlations between categorical variables and the t-test or Mann–Whitney U test were applied to compare differences in continuous variables when fulfilling or not the normality criteria, respectively. Levels of contamination between stem cell grafts and peripheral blood (PB) were tested for relevant correlations with linear and non-linear regression models. Time-to-event comparisons were performed with log-rank statistics and Cox proportional hazard regression was performed for the risk estimation of graft contamination in achieving the relevant endpoint set.

## 3. Results

### 3.1. Patients

One hundred and ninety-nine myeloma patients were enrolled in this study. The clinical characteristics of patients enrolled, according to their graft contamination status, are shown in Table 1.

Patients received different induction regimens, according to our institutional policy, with almost half of them receiving VRD. Daratumumab-based therapies (dara-VCD and Dara-VRD) were also given outside of clinical trials; thus, in total, 193 (97%) patients were given induction therapy in the real world. None of these patients received consolidation treatment, while all of them received lenalidomide maintenance (plus bortezomib every two weeks for those with high-risk cytogenetics only), according to our center protocols (i.e., no daratumumab was given as maintenance). Maintenance was given until progression or unacceptable toxicity. Only six patients, who received carfilzomib-based induction, participated in clinical trials.

The median follow-up period post ASCT was 22 months (range 1–55 months). Within this monitoring period, 15 patients showed evidence of disease progression and 13 patients succumbed; 5/13 due to a disease-unrelated cause.

### 3.2. Frequency, Phenotypic Features and Quantification of Aberrant Plasma Cells in Autologous Stem Cell Grafts

**Figure 1:**
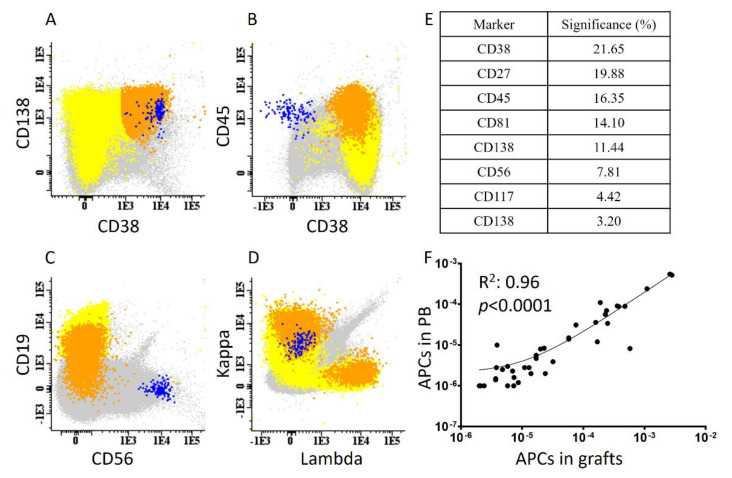
Detection of aberrant plasma cells (APCs) with next-generation flow cytometry. (**A**–**D**): Representative con+ sample with a clear subset of APCs at the level of 10^−5^ (blue). The normal plasma cell compartment is shown in orange, B cells in yellow and all other nucleated cells in grey. (**E**) Relevant significance of different markers used for the discrimination of APCs (cumulative mean values of *n* = 79 con+ cases). (**F**) Linear correlation between the percentage (%) of APCs detected in stem cell grafts and 41 matched peripheral blood (PB) samples.

Aberrant clonal plasma cells were present in 79/199 (39.7%) stem cell grafts evaluated within a median reached LOD of 3.6 × 10^−6^ (range 2–4.8 × 10^−6^). The median value of APCs in contaminated (con+) samples was 2.2 × 10^−5^ (range 2 × 10^−6^–1.2 × 10^−2^) of total nucleated cells; on a logarithmic scale, the distribution of the detection levels of APCs among con+ cases were 10% for levels higher than 10^−3^, 26.6% for detection at levels 10^−3^–10^−4^, 27.8% for levels 10^−4^–10^−5^ and 35.4% for levels lower than 10^−5^ (Figure 1A–D). Among con+ patients, the phenotype of APCs varied, but in all cases, showed the same pattern of surface and intracellular molecule expression with the APCs detected at diagnosis. In detail, the phenotype of APCs showed negativity for CD19, CD45 and CD81 in 97.5%, 90.0% and 85.0% of con+ samples, respectively. The aberrant phenotypes also included CD56 and CD117 positivity in 53% of con+ samples (concomitant expression of CD56 and CD117 in half of them), whereas CD27 was negative or weakly expressed in 90% of them. Ki67, when positive, was expressed in a very small compartment of APCs (1–5%) (Figure 1E).

We additionally tested matched PB samples from 41 con+ and 59 con− patients post mobilization. APCs were also detected in the PB, but in lower abundance as compared with the apheresis product. In particular, APCs could be detected in the PB of 29/41 (70.7%) con+ patients, and in all cases, in lower numbers than in the apheresis products (R^2^ = 0.96, *p* < 0.0001; Figure 1F). On the contrary, we found no case in which APCs were detected only in the PB and not in the matched stem cell graft.

### 3.3. Worse Responses to Induction Therapy Correlate with Higher APC Contamination Levels in Stem Cell Grafts

The presence of APC contamination in stem cell grafts was not correlated with the baseline clinical and prognostic parameters of the patients, including cytogenetics and/or the ISS staging (Table 1). The only baseline parameters that were associated with the stem cell graft contamination were the increased levels of serum β2-microglobulin (median value 3.3 mg/L for con+ vs. 2.7 mg/L for con− patients; *p* < 0.05) and the higher BM infiltration rates (median value 63% in con+ vs. 55% in con− patients; *p* < 0.05).

**Figure 2:**
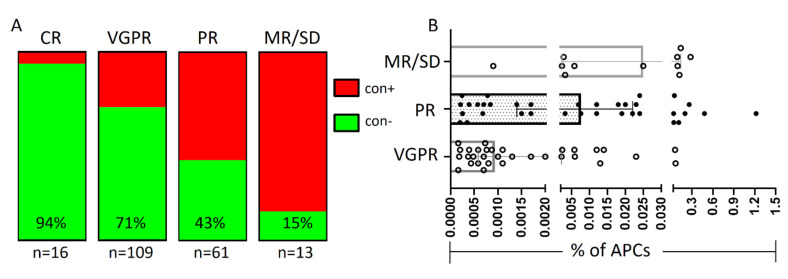
The presence of graft contamination according to the depth of response post induction treatment. Frequency of graft contamination in the various response categories (**A**) and distribution of residual myeloma burden in autologous grafts on the distinct response groups (**B**). con+, contaminated graft samples; con−, uncontaminated graft samples; CR, complete response; MR/SD, minor response/stable disease; n, number of cases in each group; PR, partial response; VGPR, very good partial response.

On the contrary, the frequency of contamination in stem cell grafts was significantly associated with the type of response to induction therapy (Figure 2A). All but one patient who achieved CR and 71% of those who achieved VGPR post induction treatment showed no contamination in their subsequent stem cell graft samples. In contrast, more than half (57%) of patients who showed PR, and the vast majority (>85%) of those who did not respond (SD) or had a minor response (MR) to initial regimens, had clear evidence of contaminated grafts. Of note, the number of residual clonal APCs in con+ grafts varied significantly among the different response categories, with an almost logarithmic increase towards worse responses (median percentage of APCs: 9.4 × 10^−6^ for patients who achieved VGPR vs. 7.6 × 10^−5^ for those who did not achieve PR vs. 8 × 10^−4^ for the MR/SD group; *p* < 0.001) (Figure 2B).

### 3.4. Uncontaminated Stem Cell Grafts Correlate with Better Responses Post ASCT

ASCT improved the response status to induction therapy in 52% of all enrolled patients; 49% of patients who achieved PR post induction improved to VGPR and 18% to CR after the completion of ASCT, whereas 41% of patients who achieved VGPR post induction improved to CR post ASCT (Table 2).

In an effort to evaluate graft contamination as an indicator for ASCT efficacy, we analyzed the effect of this parameter in each subgroup of patients with the same response status to induction treatment. Hence, for the PR group post induction, 34% of con+ patients retained the same status post ASCT and only 11% achieved CR. On the contrary, among the con− patients with PR following induction,15% retained their PR status and 27% achieved CR (Table 2). Likewise, for the VGPR group, 44% of con− patients turned to CR post ASCT vs. 34% of those with detectable APCs in their grafts. The cumulative frequency for CR achievement post ASCT was 20% for con+ and 48% for con− patients.

### 3.5. Absence of Graft Contamination Can Serve as an Early Marker of Deep Remission

To further evaluate the prognostic significance of stem cell graft contamination, we examined its impact on the time required for patients to achieve CR and MRD-negative responses post ASCT. Patients were monitored monthly for their response status, and in case of CR achievement, they were evaluated for MRD negativity with the same NGF approach and a median LOD for detecting APCs of 2.2 × 10^−6^ (range 1.9–2.6 × 10^−6^). In case of a MRD-positive result, patients were repeatedly examined for MRD negativity every 3–6 months post the prior testing based on signs of disease improvement. Overall, 180 patients were evaluated (at least once) for MRD negativity and 113 patients achieved MRD negativity within the median follow-up period post ASCT (22 months).

**Figure 3:**
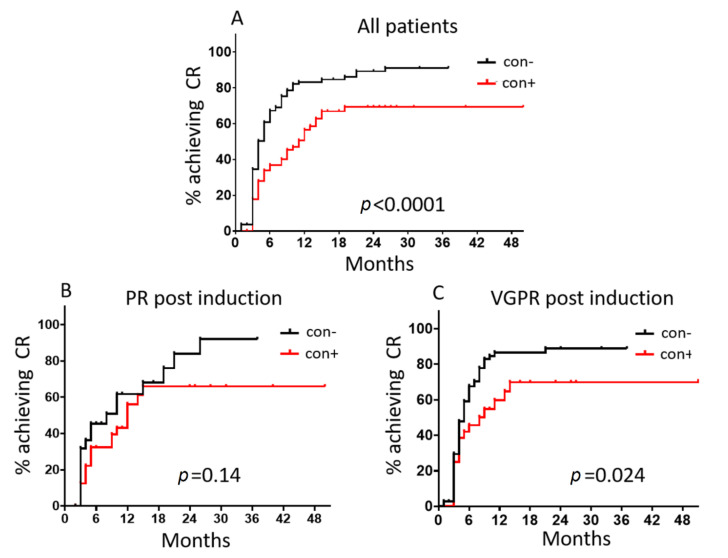
Time to CR achievement post ASCT according to graft contamination status for (**A**) all patients enrolled in the study (*n* = 199), (**B**) patients who achieved PR post induction (*n* = 61) and (**C**) patients who achieved VGPR post induction (*n* = 109). con+, contaminated graft samples; con−, uncontaminated graft samples.

In total, the median time to achieve CR was 4 and 11 months for con− and con+ patients, respectively (HR: 2.01, 95% CI: 1.44–2.81; *p* < 0.0001; Figure 3A). A similar pattern was shown in the subgroup analysis according to the depth of response before ASCT. In particular, con− patients within the PR group post induction had a median time of 8 months to achieve CR vs. 12 months for their con+ counterpart (HR: 1.59, 95% CI: 0.81–3.16; *p* = 0.14; Figure 3B). Accordingly, for the VGPR group, the median time for CR achievement for con− and con+ patients was 5 and 8 months, respectively (HR: 1.67, 95% CI: 1.06–2.63; *p* = 0.024; Figure 3C).

**Figure 4:**
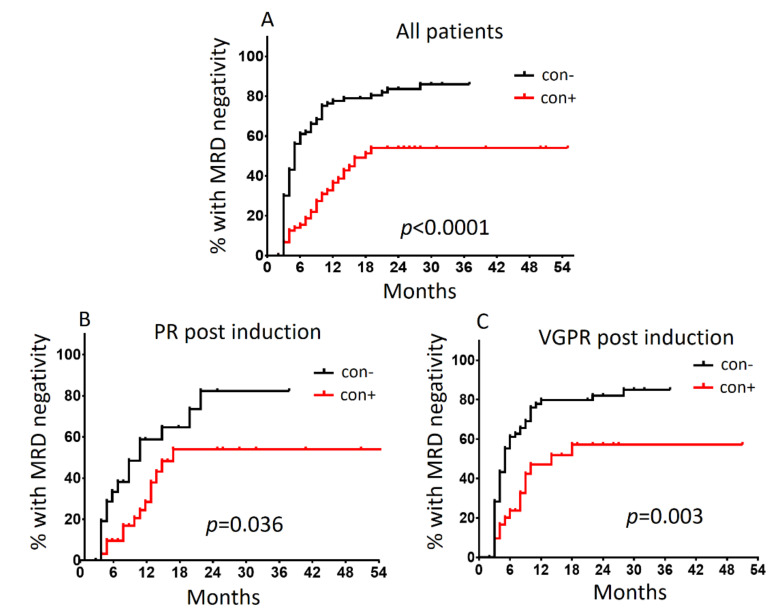
Time to MRD negativity achievement post ASCT according to graft contamination status for (**A**) all patients enrolled in the study (*n* = 199), (**B**) patients who achieved PR post induction (*n* = 61) and (**C**) patients who achieved VGPR post induction (*n* = 109). con+, contaminated graft samples; con−, uncontaminated graft samples.

The presence of APCs in the stem cell grafts had a more pronounced effect on the time required to reach deeper responses defined by MRD negativity at the LOD set. In our entire cohort, the absence of graft contamination was associated with a 2.8 greater probability of MRD negativity compared with the contaminated grafts (HR: 2.77, 95% CI: 1.91–4.01; *p* < 0.0001). The median time to reach MRD negativity was 5- and 18-months post ASCT for con− and con+ patients, respectively (*p* < 0.0001; Figure 4A). Within the PR group post induction, contamination of the graft conferred a 6-month delay in achieving MRD negativity (median 10 months for con− vs. 16 months for con+ patients; HR: 2.13, 95% CI: 0.98–4.63; *p* < 0.036). Interestingly, a more pronounced 9-month discrepancy was shown within the VGPR group (median 5 months for con− vs. 14 months for con+ patients; HR: 2.23, 95% CI: 1.36–3.65; *p* = 0.003; Figure 4B,C).

The survival analysis in terms of PFS and OS according to graft contamination status is shown in Supplementary Figure S1. The median time to progression in the median 22-month follow-up period was not reached for both groups, though there was a clear tendency for longer relapse-free periods for patients with uncontaminated grafts (2-year PFS, 92% for con− vs. 83% for con+ patients; *p* = 0.13). The OS did not show any difference in the two groups due to the very low number of patients who succumbed due to MM progression (*n* = 8) within the follow-up monitoring period.

### 3.6. Residual APCs on Grafts May Affect BM Reconstitution Post ASCT

**Figure 5:**
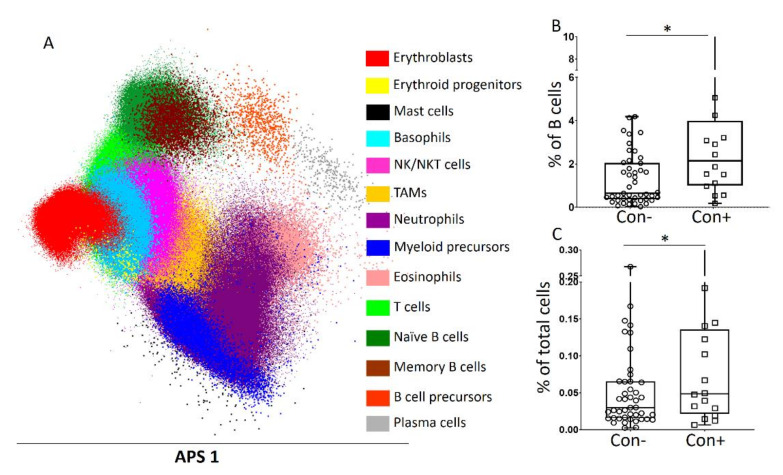
Bone marrow (BM) reconstitution post ASCT. (**A**) Individualized BM profile of a patient showing the relevant distribution of the various BM subsets. The density of each subset represents its relative abundance among total BM nucleated cells. (**B**,**C**) Memory B cell distribution between uncontaminated (con−) and contaminated (con+) graft-derived BM as expressed among total B cells (**B**) and total nucleated cells (**C**). * *p* < 0.01; TAMs, tumor-associated macrophages.

We have previously reported that the MRD status of MM patients correlates with distinct BM niche profiles [18]. To evaluate the effect of contaminated grafts on the BM reconstitution, we compared the BM signatures of con− and con+ patients who achieved CR 100 days post ASCT. The analysis revealed a significant heterogeneity in the BM subset distribution among patients; however, no significant changes between the two groups were observed. The only statistically significant difference was found in the relative abundance of memory B cells, which were found increased in the BM of con+ derived grafts (Figure 5).

## 4. Discussion

The introduction of novel anti-myeloma drugs, in parallel with the intense clinical research, has led to substantial improvement in the management of MM patients during the last decades. The majority of MM patients are now achieving deep responses with significant prolonged progression-free periods and overall survival [19]. The role of ASCT in this new drug era remains irreplaceable and constitutes the first choice for transplant-eligible newly diagnosed patients [20,21]. However, due to the multilevel heterogeneity of MM features, not all patients experience the same beneficial impact of ASCT, even among those sharing the same baseline prognostic characteristics. The presence of contamination in stem cell grafts has long been considered as a potential predictor for subsequent outcomes, but the relevant studies have utilized different detection approaches, resulting in different and often contradictory observations [10,11,12,22].

In our study, we have prospectively assessed the prognostic impact of apheresis products in a large number of newly diagnosed MM patients by means of the sensitive NGF approach [23,24]. We detected APCs in 40% apheresis samples at various levels, varying from 1% to LOD (reaching 10^−6^) of total nucleated cells. The median value of APCs was 2 × 10^−5^ and more than one third of positive samples were contaminated at levels below 10^−5^, and would have been falsely considered as negative by the application of conventional and less sensitive approaches. The presence of graft contamination did not correlate with patients’ baseline characteristics, except for a positive association with increased β2-microglobulin and BM infiltration, indicating an inadequate purging of total APCs by induction therapy and HDM for patients with initially high disease burden [11]. The phenotype of APCs in grafts had no differences compared to those detected at baseline, representing residual cells of the major clonal population detected at diagnosis. Moreover, analyses with Ki67 showed a minor fraction of positive APCs in the grafts, thus implying no treatment-related selection towards the initial proliferating counterpart.

Sensibly, the presence of graft contamination was found to directly correlate with the type of response to induction treatment. The majority of patients who achieved VGPR or better prior to ASCT had no detectable APCs in their mobilized grafts, whereas more than 70% of those with PR or inferior responses (i.e., MR or SD) showed clearly detectable clonal subsets in their grafts. The quantitative analysis also showed significant mean variations among con+ patients among the different response categories, thus reflecting the differential efficacy of induction treatment to the residual number of clonal cells. However, despite clear differences between the different response categories, there was a notable distribution of the positive APC fraction among con+ patients of the same response group. Moreover, we should point out that in a marked number of patients who achieved PR, and even in 2 patients with only MR post induction, the apheresis products were found uncontaminated, i.e., their mobilized grafts contained no APCs. This finding verifies previous observations of varying APC rates among patients with the same response status prior to ASCT [25], and confirms the existence of a prevalence of normal plasma cells in mobilized stem cell harvests of MM patients [26].

Although the administration of ASCT may substantially improve treatment responses to induction therapy, there is sufficient evidence that the depth of response to induction regimens prior to ASCT is predictive of different PFS achieved post ASCT. This is highly linked to the different frequencies of response improvement achieved post ASCT, related to the initial response status [21,27,28,29,30]. For example, in our cohort, 41% of patients with an initial VGPR turned to CR post ASCT, compared with only 18% of patients with an initial PR post induction. Whether or not MM patients will improve their response status after ASCT is not predefined, but our results highlight graft contamination as a useful biomarker with predictive value. Indeed, the possibility of a CR improvement on day 100 post ASCT was 1.8-fold higher in con− patients who showed PR/VGPR post induction and 2.5-fold higher when considering PR patients alone.

Most importantly, though, we showed that the pre-ASCT evaluation of graft contamination could be predictive of deep remissions. In time-to-event analyses, the presence of APCs in stem cell grafts was correlated with longer periods taken to achieve CR and MRD negativity. The correlation was attained when analyses were adjusted within the same response category, clearly subdividing patients into two groups with a diverse risk of not reaching or achieving delayed deep responses. Of note, this correlation was stronger when we set MRD negativity and not CR as the clinical endpoint, thus implying that the absence of graft contamination could be a strong predictor of deep and lasting remissions. MRD negativity is a distinct and powerful independent prognostic factor in MM, which may overcome baseline prognostication by ISS and/or cytogenetics [31,32], and is currently considered as the main or secondary endpoint in several ongoing trials [33,34]. Therefore, the identification of biomarkers capable of an early prediction of MRD negativity is of utmost significance for the clinical management of MM patients and may confer significant surrogate information on modern tailored MRD-driven approaches [34,35].

The BM microenvironment has a key supportive role for myeloma growth and progression, with distinct underlying biology at different disease stages [13,15,36,37]. Various BM subsets interact constantly and dynamically with myeloma cells and contribute to the complex immunomodulatory functions induced, as well as drug refractoriness [38,39]. Therefore, the elucidation of the exact molecular interactions within the BM and the identification of unique immune signatures that may have clinical utility in terms of prognostication and/or drug efficacy prediction is a very active field in MM research [40]. In this context, we recently reported on unique BM signatures that are predictive of different responses to the same VRD induction therapy [18]. Moreover, we showed that, despite the apparent innate heterogeneity in the BM subset distribution post ASCT, patients with the same MRD status (positive vs. negative) shared commonmicroenvironmental features that allowed for their efficient clustering in the two groups [18]. In order to assess whether the presence of APCs in grafts could result in a different BM profile, we evaluated the niche composition on day 100 post ASCT. Our analysis revealed high variations among patients’ profiles; nevertheless, a significantly higher prevalence of memory B cells among con+ derived BM was detected. This finding is in agreement with our previous observations that showed a higher abundance of this particular B cell subset in the BM of MRD-positive MM patients [18]. The exact implication of the various BM subsets in MRD biology and disease progression is yet to be clarified, though it was proposed that a skewed B cell subset ratio has an impact on differential clinical outcomes, which is probably associated with long-term disease control [40].

Our study has specific limitations regarding the heterogeneous treatment modalities administered to the patients. We should note that approximately half of the patients enrolled received the same VRD induction treatment. Although the different induction regimens were balanced in the two groups, the drug regimen was positively correlated with the absence of contamination only in patients treated with Dara VRD (11.6% of total patients). Moreover, the clinical evaluation of graft contamination was applied on the basis of the common response status, which likely eliminates any potential bias. Importantly, the tendency of worse prediction for con+ patients remained significant when we adjusted analysis on the basis of the same or similar induction treatment (data not shown). The post-ASCT treatment was common in all but six patients who participated in clinical trials and received different consolidation or maintenance therapies. All other patients were treated according to institutional “real world” induction regimens followed by ASCT and lenalidomide maintenance until progression or unacceptable toxicity; therefore, the potential bias was drastically reduced. Another limitation is the short follow-up monitoring post ASCT, which does not allow validation of the clinical impact of graft contamination in terms of PFS and OS, due to the low number of patients who relapsed and succumbed during the study period. Thus, although there is a trend for a better PFS in the absence of graft contamination, a longer follow-up period is needed to draw final conclusions for a possible PFS advantage in these patients.

To the best of our knowledge, this is the largest prospective study evaluating the clinical impact of stem cell graft contamination with the highly sensitive NGF approach. Our results highlight that the early assessment of graft contamination is a negative prognostic biomarker that is related to delayed deep remissions and a more challenging MRD negativity achievement. The design of large prospective studies on transplant-eligible patients receiving the same regimens and a long follow-up monitoring period are needed to establish graft contamination, of even marginal residual disease, as an early predictive and/or prognostic biomarker with robust clinical utility.

## 5. Conclusions

ASCT remains the most beneficial option for transplant-eligible newly diagnosed MM patients, though response improvement and clinical outcomes post ASCT remain heterogeneous. As novel biomarkers with a strong predictive value are needed for the early recognition of those patients with a higher risk of progression, the evaluation of stem cell graft contamination with sensitive approaches may serve as a predictive factor post ASCT, which may clearly stratify patients into distinct risk categories according to their potential to progress. In this context, tailored therapeutic decisions would be made.

## Figures and Tables

**Table 1:** Association of ASCT contamination with baseline clinical features of patients with MM.

Clinical Parameters	Con− (*N* = 120)	Con+ (*N* = 79)	*p* Value
Age, years	55.9 (38–66) *	57.3 (35–66)	ns
Male sex (%)	51/80 (63.8%)	26/48 (54.2%)	ns
Hemoglobin, g/dL	11.7 (7.5–16.5)	11.3 (5.8–16.7)	ns
Platelet count, ×10^9^/L	253 (73–805)	249 (55–490)	ns
Neutrophils, counts/μL	3800 (1100–21,700)	3400 (1000–11,100)	ns
Serum Albumin, g/dL	4.0 (0.98–5.3)	4.0 (2.2–5.3)	ns
Serum Creatinine, mg/dL	0.81 (0.42–10.7)	0.87 (0.41–12.5)	ns
Serum Calcium, mg/dL	9.5 (6.1–17)	9.4 (7.7–14)	ns
Serum β2-microglobulin, mg/L	2.7 (1.3–28.1)	3.3 (1.2–24.8)	0.046
Serum LDH, U/L	162 (71–476)	173 (51–557)	ns
BM infiltration, %	55 (3–100)	63 (10–100)	0.036
Osteolytic bone disease (%)	89/120 (74.2%)	62/79 (79.5%)	ns
ISS stage			ns
I	71/119 (59.7%)	36/78 (46.2%)	
II	30/119 (25.2%)	23/78 (29.5%)	
III	18/119 (15.1%)	19/78 (24.4%)	
High risk cytogenetics (t(4;14), t(14;16) and/or del(17p13) and/or +1q)	27/106 (25.5%)	14/66 (21.1%)	ns
Heavy chain			ns
IgA	25/120 (20.8%)	14/79 (17.7%)	
IgG	72/120 (60%)	48/79 (60.8%)	
IgD	1/120 (0.8%)	1/79 (1.3%)	
Light chain only	20/120 (16.7%)	15/79 (18.9%)	
Non-secretory	1/120 (0.8%)	1/79 (1.3%)	
IgA + IgG	1/120 (0.8%)	-	
Kappa Light chain	71/120 (59.2%)	53/79 (67.1%)	ns
Induction treatment			
VCD	21 (17.5%)	24 (30.3%)	ns
VTD	9 (7.5%)	8 (10.1%)	ns
VRD	57 (47.5%)	37 (46.8%)	ns
DaraVCD	8 (6.6%)	6 (7.6%)	ns
DaraVRD	22 (18.3%)	1 (1.3%)	0.0001
Others (KD, KRD, DaraKD, IsaKRD)	3 (2.5%)	3 (3.8%)	ns

* range in brackets; BM, bone marrow; con+, contaminated graft; con−, uncontaminated graft; C, cyclophosphamide; Dara, daratumumab; D, dexamethasone; Isa, isatuximab; ISS, international staging system; K, calfizomib; LDH, lactate dehydrogenase; ns, not significant; R, lenalidomide; T, thalidomide; V, bortezomib.

**Table 2:** Response improvement post ASCT according to stem cell graft contamination status.

	Contaminated Grafts				Uncontaminated Grafts				*p* Value
	Total	PR Post ASCT	VGPR Post ASCT	CR Post ASCT	Total	PR Post ASCT	VGPR Post ASCT	CR Post ASCT	
MR/SD post induction *n* = 13 *	11	5/11 (45%)	4/11 (36%)		2			1/2 (50%)	na
PR post induction *n* = 61 **	35	12/35 (34%)	17/35 (49%)	4/35 (11%)	26	4/26 (15%)	13/26 (50%)	7/26 (27%)	ns
VGPR post induction *n* = 109	32		21/32 (66%)	11/32 (34%)	77		43/77 (56%)	34/77 (44%)	ns
CR post induction *n* = 16	1			1	15			15 ***	na
Total CR rate	79			16/79 (20%)	120			57/120 (48%)	0.0001

* Two patients showed progressive disease and one patient expired during ASCT due to infection;** two patients showed progressive disease and two patients expired during ASCT; *** three patients turned to stringent CR post ASCT; ASCT, autologous stem cell transplantation; CR, complete response; MR/SD, minor response/stable disease; na, non-applicable; ns, not significant; PR, partial response; VGPR, very good partial response.

## Data Availability

The results of the study are available upon request from the corresponding authors.

## Supplementary Materials

The following are available online at https://www.mdpi.com/article/10.3390/cancers13164047/s1. Figure S1: Progression free survival (PFS) and overall survival (OS) post autologous stem cell transplantation according to the presence (con+) or absence (con-) of APC contamination in the apheresis product.
