# Supplementary material for: Aberrant Plasma Cell Contamination of Peripheral Blood Stem Cell Autografts, Assessed by Next-Generation Flow Cytometry, Is a Negative Predictor for Deep Response Post Autologous Transplantation in Multiple Myeloma; A Prospective Study in 199 Patients
Source: Cancers (Basel). 2021 Aug 11;13(16):4047. doi: 10.3390/cancers13164047 (PMC8391595; doi:10.3390/cancers13164047)
Supplement: Supplementary file 1 [file cancers-13-04047-s001.zip › cancers-1273358-supplementary.pptx]

## Slide 1
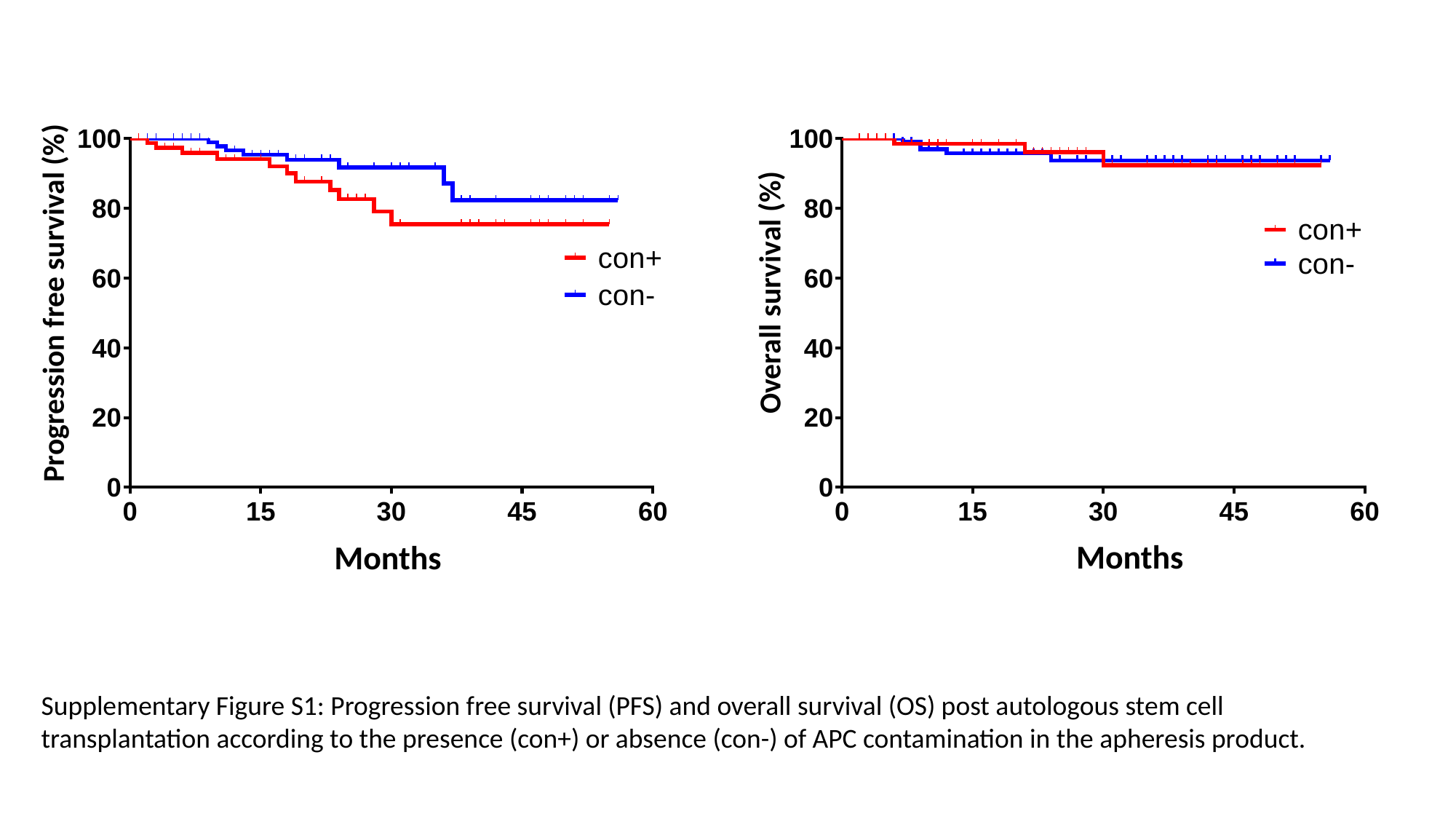

Progression free survival (%)
Months
Overall survival (%)
Months
Supplementary Figure S1: Progression free survival (PFS) and overall survival (OS) post autologous stem cell transplantation according to the presence (con+) or absence (con-) of APC contamination in the apheresis product.
